# Supplementary material for: HPOSS: A hierarchical portfolio optimization stacking strategy to reduce the generalization error of ensembles of models
Source: PLoS One. 2023 Aug 31;18(8):e0290331. doi: 10.1371/journal.pone.0290331 (PMC10470931; doi:10.1371/journal.pone.0290331)
Supplement: Attachment — Submitted filename: Reply_to_Reviewers__HPOSS___PlosOne__Rev1_ (1).pdf [file pone.0290331.s001.pdf]

Authors’ Response to Reviews of

HPOSS: A hierarchical portfolio optimization stacking strategy to reduce the generalization error of ensembles of models

Ozelim et al.
PLoSOne, PONE-D-23-08481

RC: ***Reviewers’ Comment***, AR: Authors’ Response, ☐ Manuscript Text

Dear Prof. Dr. Argimiro Arratia, Academic Editor of PLoSOne

We thank the valuable comments provided by the reviewers and also by the Academic Editor. A complete reply to each of the questions raised is hereby presented. We hope you find our manuscript suitable for publication and look forward to hearing from you in due course.

1. Academic Editor

RC: ***Please address both reviewer’s comments, although I would add certain provisos: -regarding the listing of scikit-learn library (p6) is not completely unnecessary but certainly useless for the reader the way it is. It should be more instructive to mention the model by classes (linear regressions all available, trees models, neural networks, etc) and which constitute emsembles. This list can then be shorten or substitute by a summary.***

AR: We thank the Editor for pointing out this issue. We have rewritten the listing of scikit-learn library algorithms in a much shorter way as follows:

> This way, in order to enforce diversity, a total of forty algorithms gathered into ten model classes were considered, namely: **ensemble** (which try to enhance the overall generalizability and robustness while compared to a single predictor by merging the forecasts of multiple predictors generated using a particular learning method - ‘AdaBoostRegressor’, ’BaggingRegressor’, ‘ExtraTreesRegressor’, ‘GradientBoostingRegressor’, ‘HistGradientBoostingRegressor’, ‘RandomForestRegressor’); **linear_model** (operate under the assumption that the desired output will be a result of combining the input features in a linear manner - ‘BayesianRidge’, ‘ElasticNet’, ‘ElasticNetCV’, ’GammaRegressor’, ‘HuberRegressor’, ‘Lars’, ‘LarsCV’, ‘Lasso’, ‘LassoCV’, ‘LassoLars’, ‘LassoLarsCV’, ‘LassoLarsIC’, ‘LinearRegression’, ‘LinearSVR’, ‘OrthogonalMatchingPursuit’, ‘OrthogonalMatchingPursuitCV’, ‘PassiveAggressiveRegressor’, ‘PoissonRegressor’, ’QuantileRegressor’,’RANSACRegressor’, ‘Ridge’, ‘RidgeCV’, ‘SGDRegressor’, ‘TweedieRegressor’); **tree** (acquire simple decision rules from the provided input data to construct a model capable of predicting the target variable’s value - ‘DecisionTreeRegressor’, ‘ExtraTreeRegressor’); **dummy** (make predictions using uncomplicated rules, serving as basic benchmarks for comparison with other regression models – ‘DummyRegressor’); **gaussian_process** (also known as kriging algorithms - ’GaussianProcessRegressor’); **neighbors** (assign a numerical value to a given point by calculating the mean(or other summarizing function) of the values from its closest neighbors - ’KNeighborsRegressor’); **kernel_ridge** (combines ridge with the kernel trick -

’KernelRidge’); **neural_network** (simple multi-layer perceptron regressor - ’MLPRegressor’); **svm** (support vector machine based models - ’NuSVR’, ’SVR’) and **compose** (meta-estimator to regress on a transformed target - ’TransformedTargetRegressor’);

RC: ***- describe the parameters considered for tuning***

AR: In the first version of the manuscript, we had pointed out that:

> These techniques did not consider manual hyperparameter tuning because, when combined, even weak predictors can result in a robust global prediction of the output variables [35].

We believe that such statement was not clear enough to indicate that no hyperparameter tuning was performed by the authors, only the ones already built in the *scikit-learn* algorithms. In order to account for that, the paragraph cited now reads:

> When dealing with a vast list of machine learning models, it becomes evident that hyperparameter tuning may not be imperative [67]. This is due to the fact that when weak predictors are aggregated and combined, they possess the capability to produce robust estimates. By harnessing the collective knowledge of multiple models, even those with relatively modest individual predictive capabilities, the resultant ensemble is able to offset individual shortcomings and attain more dependable and precise predictions. As a result, the emphasis transitions from fine-tuning hyperparameters for individual models to the creation and combination of diverse models, thereby enhancing the ensemble’s overall performance and capacity to generalize. This is precisely the focus of the present paper.

RC: ***- provide the data (one reviewer is not clear about it , and it is a requirement of PLOSONE to have data)***

AR: We believe the reviewer is referring to the dataset used to calibrate the metamodels which are then filtered and weighted. Thus, we uploaded such dataset to Zenodo and have cited its existence within the manuscript as:

> The dataset can be found in [88], where a *.h5* file is present. The file contains a dataset called "Calibrations_LHS", which consists of a 80×5 numpy array of float numbers corresponding to $d_1(m)$,$d_2(m)$, $d_3(m)$, $d_4(m)$ and $\zeta_{max}(Pa)$, where d_i, $i = 1, ..., 4$ are dimensions (in meters) of the I-beam and ζ_{max} is the maximum bending stress (in Pascals) developed in such beam.

RC: ***- Distance metrics among assets (p 10) it is implied this is amomg the financial time series of prices or returns, for which I am not particularly fond of Pearson distance metric as it assumes a linear relation among the variables. I’ll rather recommend instead Spearman or Kendall which captures comovements or much better the distance correlation, which characterizes independence. In the realm of clustering time series these latter are more effective than Pearson. See for example a study I did on this some time ago: A. Arratia & Marti Renedo (2016). Clustering of exchange rates and their dynamics under different dependence measures (PDF). In: ECML-PKDD. Proc. 1st Workshop MIDAS (MIning DAta for financial applicationS). September 19, 2016 - Riva del Garda, Italy. CEUR-WS, v. 1774, pp 17-28. https://ceur-ws.org/Vol-1774/***

AR: We agree with the Academic Editor. Previously, we had considered those correlation coefficients to build distance metrics, but ended up not including their application in the final version paper. Now, we included both correlation measures and discussed their applicability in the HPOSS context. The following paragraphs and figures haven been added to the manuscript (figures are correctly sized in the manuscript):

- Kendall's τ correlation distance

Similarly to the Pearson correlation distance, it is possible to consider Kendall's τ rank correlation to build a distance metric τ_{ij} between pairs of assets. Literature reveals that this correlation metric better captures co-movements, while compared to Pearson correlation, especially in the realm of clustering time series [55].This way, a $m \times m$ distance matrix, whose elements are $D_{\tau,ij} = \sqrt{(1 - \tau_{ij})/2}$, can be defined.

- Spearman's ρ correlation distance

Literature also indicates that Spearman's ρ, ρ_S, has a good performance when used to cluster financial assets' returns [55] and, therefore, can be used to build a distance metric $\rho_{s,ij}$ between pairs of assets. This way, a $m \times m$ distance matrix, whose elements are $D_{\rho,ij} = \sqrt{(1 - \rho_{s,ij})/2}$, can be defined.

[...]

For the DBHT algorithm, the similarity measure chosen was the generalization error similarity. The clusters obtained from running the DBHT algorithm with different distance metrics (Pearson, Kendall' τ and Spearman's ρ correlation distances, approximate ratio correlation distance and approximate normal correlation distance) were also evaluated, and the clusters obtained for the Pearson, Kendall' τ and Spearman's ρ correlation distances are presented in Figures 5 to 7.

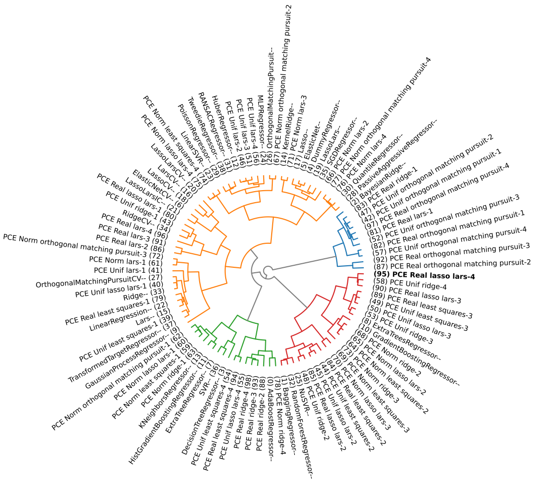

Figure 5: **Radial dendrogram obtained from DBHT algorithm with Pearson correlation distance and generalization error similarity**

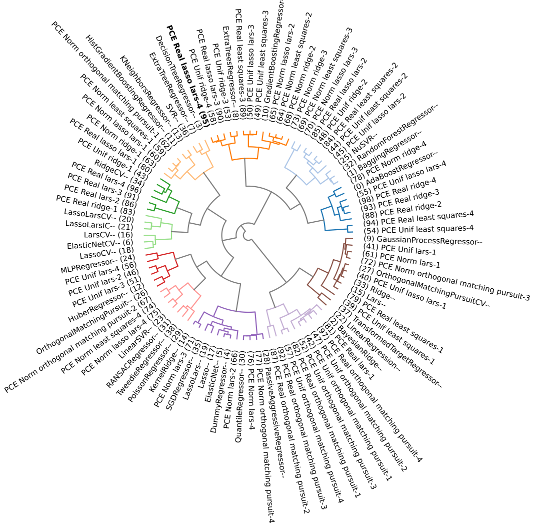

Figure 6: **Radial dendrogram obtained from DBHT algorithm with Kendall' τ correlation distance and generalization error similarity**

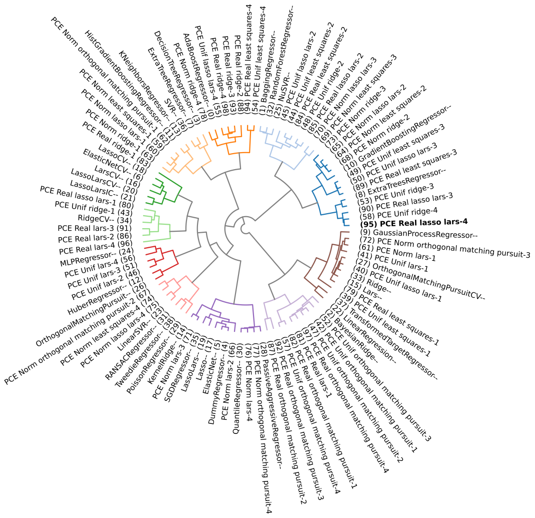

Figure 7: **Radial dendrogram obtained from DBHT algorithm with Spearman's ρ correlation distance and generalization error similarity.**

The analysis of Figures from 5 to 7 indicate that the DBHT algorithm with both Kendall' τ and Spearman's ρ correlation distance metrics provided the same clustering result (same models clustered together with the "PCE Real Lasso Lars-4" model). Moreover, with the use of Pearson correlation distance metric, the DBHT algorithm provided a bigger cluster which contains not only the models clustered by DBHT using the other distance metrics, but also extra models. This indicates that if the

Pearson correlation is used, a looser clustering process is carried out, which ends up including more models in the final cluster.

In the present paper, we seek to use the DBHT algorithm to filter among a large number of candidate models. Therefore, while we intend to eliminate a good share of uncorrelated models, we still want to have a good number of algorithms in the final cluster. Therefore, we chose the Pearson correlation distance metric to carry out the study case. Depending on the specific application envisioned, the reader may, on the other hand, choose a tighter clustering scheme, by selecting either Kendall' τ or Spearman's ρ correlation distance metrics together with the DBHT algorithm.

RC: ***- Regarding robust optimization (Eq 35) I wonder if one can simplify the maxmin problem to one of maximization: in vectorize notation (with d=delta): x(p+ dz) = xp + xdz >= xp - ||xd||||z|| (by Cauchy-Schwarz) >= xp - ||xd||, since ||z|| < 1 hence the problem becomes maximize_x: xp - ||xd||, subject to sum x_i = 1, x > 0 so there is no need to sample random variable Z... is this correct?***

AR: This question is quite interesting. By checking equation (25) in the work of Bertsimas & Sim (2004) - "The Price of Robustness", it is possible to see that the maxmin optimization approach was written in terms of a maximization problem subjected to inequality constraints. We believe the maxmin approach was chosen in the *Rsome* python package due to its generalizability capacity (especially with respect to other uncertainty sets). We also believe that the direct application of the Cauchy-Schwarz inequality, as suggested by the Academic Editor, may not be always ideal, since we are dealing with random variables instead of deterministic scalar quantities. The AE may check that his proposed approach ends up considering only the lower boundary of the uncertain set for asset returns (worst-case scenario: p - delta), being more restrictive than the maxmin approach considered. This is a more conservative portfolio, while compared to the one obtained in the maxmin approach. If we chose, for example, to deal with expected values of the returns, a different simplification would be needed. This comes from the fact that Let $X_1, ...X_n$ be IID random variables (which could be assumed in some cases in the cotext of the robust optimization algorithm), then $\min\{X_1, \ldots, X_n\}$ is also a random variable (and not the actual lower end of the random variables' supports). This is, for sure, an interesting topic which may be further addressed in optimization-related papers.

2. Reviewer #1

RC: ***The evaluation and construction methodology for a bagging (or ensemble) method presented in the paper is interesting and innovative, making it suitable for submission. The paper covers various aspects of machine learning concepts, such as estimating generalization error through cross-validation, as well as structural reliability concepts like the definition of limit state functions. It makes the paper very complete and at the same time not especially clear. For PLOS ONE, I recommend a major revision, not in terms of content, but to enhance the overall clarity of the message. Here are some suggestions for the author prior to publication: - Consider different assumptions regarding the readers' existing knowledge.***

AR: We thank the reviewer for pointing that the structure of the paper needed to be enhanced. We also appreciate the suggestions to simplify sections which contained widely known information.

RC: ***- Detailed information about the scikit-learn library (p6) is unnecessary.***

AR: We thank the reviewer for pointing out that the text was not useful as it was. We have rewritten the listing of scikit-learn library algorithms in a much shorter and more informative way as follows:

> This way, in order to enforce diversity, a total of forty algorithms gathered into ten model classes were considered, namely: **ensemble** (which try to enhance the overall generalizability and robustness while compared to a single predictor by merging the forecasts of multiple predictors generated using a particular learning method - 'AdaBoostRegressor', 'BaggingRegressor', 'ExtraTreesRegressor', 'GradientBoostingRegressor', 'HistGradientBoostingRegressor', 'RandomForestRegressor'); **linear_model** (operate under the assumption that the desired output will be a result of combining the input features in a linear manner - 'BayesianRidge', 'ElasticNet', 'ElasticNetCV', 'GammaRegressor', 'HuberRegressor', 'Lars', 'LarsCV', 'Lasso', 'LassoCV', 'LassoLars', 'LassoLarsCV', 'LassoLarsIC', 'LinearRegression', 'LinearSVR', 'OrthogonalMatchingPursuit', 'OrthogonalMatchingPursuitCV', 'PassiveAggressiveRegressor', 'PoissonRegressor', 'QuantileRegressor','RANSACRegressor', 'Ridge', 'RidgeCV', 'SGDRegressor', 'TweedieRegressor'); **tree** (acquire simple decision rules from the provided input data to construct a model capable of predicting the target variable's value - 'DecisionTreeRegressor', 'ExtraTreeRegressor'); **dummy** (make predictions using uncomplicated rules, serving as basic benchmarks for comparison with other regression models – 'DummyRegressor'); **gaussian_process** (also known as kriging algorithms - 'GaussianProcessRegressor'); **neighbors** (assign a numerical value to a given point by calculating the mean(or other summarizing function) of the values from its closest neighbors - 'KNeighborsRegressor'); **kernel_ridge** (combines ridge with the kernel trick - 'KernelRidge'); **neural_network** (simple multi-layer perceptron regressor - 'MLPRegressor'); **svm** (support vector machine based models - 'NuSVR', 'SVR') and **compose** (meta-estimator to regress on a transformed target - 'TransformedTargetRegressor');

RC: ***Explanations of the loss function, limit-state function, polynomial chaos expansion, and Kolmogorov test are not crucial to the core of the paper, as they are well-known in various communities. References are provided for interested readers.***

AR: The topics covering the limit-state function, the polynomial chaos expansion, and the Kolmogorov test have been considerably summarized in the paper, as requested. In particular, we understand that the explanations about the formal definition of the generalization error and loss function is of interest not because it is a new concept, but because we use it to discuss Theorem 1. Therefore, we humbly understand it is important to keep such explanations.

RC: ***- On the other hand, calibration, being a fundamental step in the proposed procedure, could benefit from more detailed explanations, as it may vary across different communities such as machine learning, deep learning, and reliability.***

AR: In the first version of the manuscript, we had pointed out that:

> These techniques did not consider manual hyperparameter tuning because, when combined, even weak predictors can result in a robust global prediction of the output variables [35].

We believe that such statement was not clear enough to indicate that no hyperparameter tuning was performed by the authors, only the ones already built in the *scikit-learn* algorithms. In order to account for that, the paragraph cited now reads:

> When dealing with a vast list of machine learning models, it becomes evident that hyperparameter tuning may not be imperative [67]. This is due to the fact that when weak predictors are aggregated and combined, they possess the capability to produce robust estimates. By harnessing the collective knowledge of multiple models, even those with relatively modest individual predictive capabilities, the

resultant ensemble is able to offset individual shortcomings and attain more dependable and precise predictions. As a result, the emphasis transitions from fine-tuning hyperparameters for individual models to the creation and combination of diverse models, thereby enhancing the ensemble's overall performance and capacity to generalize. This is precisely the focus of the present paper.

RC: ***- Include explicit results of the proposed approach at multiple stages.***

AR: We thank the reviewer for the suggestion. We included a new discussion on the use of other correlation distance metrics (as suggested by the Academic Editor) which presents an intermediate result of the proposed approach (the first step, which is clustering the algorithms worth stacking). We also included Figure 10 in the paper, to better illustrate the results from the second methodological step (which is finding the weights of the stacked models' predictions). Besides, we also added two paragraphs quantitatively indicating the power of the filtering process (please refer to the next issue presented by you). Please refer to the fourth response presented to the AE in this rebuttal for the new additions on correlation metrics. For the new Figure illustrating the weights, the manuscript now reads (figure in correct size within the manuscript):

In special, Figure 9 shows that “MinADWeights" present an unbalanced scheme where too much importance was given to the “PCE Real lasso lars-2" model. This illustrates how a pure optimization approach may lead to less interpretable weights. Figure 10, on the other hand, presents a heatmap for the methods whose maximum weights were lower than 0.3.

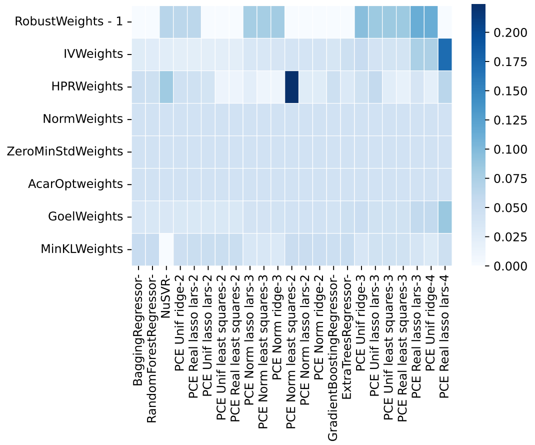

Figure 10: **Heatmap of weights.**

RC: ***- The current example solely focuses on stacking an excessive number of models, but it would be beneficial to explore what seems to be a statistically sound approach that combines information from fewer models in a more frugal manner (less diversity, fewer models).***

AR: In the first version of the manuscript, we believe we were not clear about the filtering process of the massive numbers of models considered. Thus, we included the following paragraphs along the Study Case to make things clearer:

> [...]
>
> Overall, a total of 124 models were trained using the dataset described: 40 Machine Learning models and 84 PCE-based models (3 types of random variables considered fitted using 7 least square algorithms and up to fourth order expansions, i.e., $3 \times 7 \times 4 = 84$). Among those, 99 models could be fitted without convergence and other numerical issues.
>
> [...]
>
> By considering the DBHT algorithm with Pearson correlation distance and generalization error similarity, from the 99 fitted models, a subgroup of 23 was selected to be stacked. This represents a reduction of about 77% in the total number of models, representing a good filtering scheme which still preserves diversity.
>
> [...]

RC: ***In conclusion, the proposed methodology is interesting and offers a unique perspective. However, the paper's details, in my humble opinion, do not effectively convey the core content of the approach to the reader. Additionally, the choice of application does not solidify the confidence in the method for the user. I recommend to revisit the structure of the paper to really expose the core contribution.***

AR: We thank the reviewer for pointing out that the structure of the paper demanded amendments in order to properly state our contributions. We have completely restructured the paper and hope that the reviewer finds it suitable for publication.

3. Reviewer #2

RC: ***A novel stacking strategy will be presented in this paper. This new strategy results from reinterpreting the model selection process based on the generalization error. For the first time, it will be demonstrated that this problem can be translated into a well-studied financial problem: portfolio management and optimization. The subject addressed is interesting and within the scope of the PONE. After solving the following issues, the manuscript could be possible for publication. - Authors should add quantity results to the abstract.***

AR: We thank the reviewer for pointing this flaw in the abstract. We have included some quantitative results in it, which now reads as (new additions highlighted):

> Surrogate models are frequently used to replace costly engineering simulations. A single surrogate is frequently chosen based on previous experience or by fitting multiple surrogates and selecting one based on mean cross-validation errors. A novel stacking strategy will be presented in this paper. This new strategy results from reinterpreting the model selection process based on the generalization error. For the first time, this problem is proposed to be translated into a well-studied financial problem: portfolio management and optimization. In short, it is demonstrated that the individual residues calculated by leave-one-out procedures are samples from a given random variable ϵ_i, whose second non-central moment is the i-th model's generalization error. Thus, a stacking methodology based solely on evaluating the behavior of the linear combination of the random variables ϵ_i is proposed. At first, several surrogate models are calibrated. The Directed Bubble Hierarchical Tree (DBHT) clustering algorithm

> is then used to determine which models are worth stacking. The stacking weights can be calculated using any financial approach to the portfolio optimization problem. This alternative understanding of the problem enables practitioners to use established financial methodologies to calculate the models' weights, significantly improving the ensemble of models' out-of-sample performance. A study case is carried out to demonstrate the applicability of the new methodology. **Overall, a total of 124 models were trained using a specific dataset: 40 Machine Learning models and 84 Polynomial Chaos Expansion models (which considered 3 types of base random variables, 7 least square algorithms for fitting the up to fourth order expansion's coefficients). Among those, 99 models could be fitted without convergence and other numerical issues. The DBHT algorithm with Pearson correlation distance and generalization error similarity was able to select a subgroup of 23 models from the 99 fitted ones, implying a reduction of about 77% in the total number of models, representing a good filtering scheme which still preserves diversity**. Finally, it has been demonstrated that the weights obtained by building a Hierarchical Risk Parity (HPR) portfolio perform better for various input random variables, indicating better out-of-sample performance. In this way, an economic stacking strategy has demonstrated its worth in improving the out-of-sample capabilities of stacked models, which illustrates how the new understanding of model stacking methodologies may be useful.

RC: ***- The literature review is too old, new studies should be reviewed. You can cite to following studies: Morshed-Bozorgdel, A., Kadkhodazadeh, M., Valikhan Anaraki, M., & Farzin, S. (2022). A novel framework based on the stacking ensemble machine learning (SEML) method: application in wind speed modeling. Atmosphere, 13(5), 758. Shi, J., Li, C., & Yan, X. (2023). Artificial intelligence for load forecasting: A stacking learning approach based on ensemble diversity regularization. Energy, 262, 125295.***

AR: We thank the reviewer for suggesting these two interesting studies. They have been cited and incorporated into the paper, as seen below:

> Literature reveals that ESM approaches can considerably increase the modelling accuracy, as was in the case of wind speed modelling [22]. In the latter work, the authors proposed a novel framework based on the stacking ensemble machine learning method. They considered eleven base machine learning algorithms in several categories (neuron based categories, kernel based, tree based, gradient boosted, least squares boost, curve based, regression based and hybrid algorithm based) as a first step to then apply a least squares boost using the output of the base algorithms.
>
> In another paper, other authors [23] statistically analyze the generalization error of ensemble learning to assess base-learners' diversity. In their model, they first perform an input feature selection procedure based on various tree-based embedded methods. The candidate models to be stacked (in their case, passed on to a second layer meta-learner) are then selected based on diversity regularization and individual learning capability. Those authors also apply information theory and standard hierarchical clustering algorithms to quantitatively assess the dissimilarity degree among candidate models by analyzing their error distributions. Their stacking ensemble framework employed a two layer-meta learning leave-one-out procedure.

RC: ***- The structure of the article is not interesting. An article should include the parts of the abstract, introduction, materials and methods, results and discussion, and conclusion. This study should be revised according to the mentioned structure.***

AR: We thank the reviewer for pointing out that the structure of the paper demanded amendments in order to

properly state our contributions. We have completely restructured the paper and hope that the reviewer finds it suitable for publication.

RC: ***- The statistical characteristics of case studies should be presented in one table.***

AR: We are not fully confident to have understood what the esteemed reviewer indicates as statistical characteristics. On the other hand, several new inclusions were made into the study case to better characterize the steps and their results. We hope the reviewer finds the new version sufficiently informative.

RC: ***- More figures such as the convergence curve of optimization algorithms should be added to the manuscript.***
- Are parameters of the introduced algorithm selected by sensitivity analysis? Please add the results of the sensitivity analysis.

AR: In the first version of the manuscript, we had pointed out that:

> These techniques did not consider manual hyperparameter tuning because, when combined, even weak predictors can result in a robust global prediction of the output variables [35].

We believe that such statement was not clear enough to indicate that no hyperparameter tuning was performed by the authors, only the ones already built in the *scikit-learn* algorithms. In order to account for that, the paragraph cited now reads:

> When dealing with a vast list of machine learning models, it becomes evident that hyperparameter tuning may not be imperative [67]. This is due to the fact that when weak predictors are aggregated and combined, they possess the capability to produce robust estimates. By harnessing the collective knowledge of multiple models, even those with relatively modest individual predictive capabilities, the resultant ensemble is able to offset individual shortcomings and attain more dependable and precise predictions. As a result, the emphasis transitions from fine-tuning hyperparameters for individual models to the creation and combination of diverse models, thereby enhancing the ensemble's overall performance and capacity to generalize. This is precisely the focus of the present paper.

RC: ***- Authors should add quantity results in the conclusion.***

AR: We thank the reviewer for indicating this issue with the conclusion section. Some quantitative results have been added to it, improving its quality. The updated paragraphs can be seen below:

> A two-step methodology is proposed: first, models are calibrated, and, based on their leave-one-out residues, a subset of surrogates is chosen to be stacked. To illustrate the application of the methodology, a study case was performed and revealed that, among 99 fitted models, the Directed Bubble Hierarchical Tree - DBHT - algorithm (with a generalization error similarity matrix and a Pearson correlation distance matrix) was able to cluster a small subgroup of only 23 models that were worth staking. This represents a reduction of about 77% in the total number of models, representing a good filtering scheme which still preserved analytical diversity.
>
> By considering a probability of failure example, a custom metric was defined to quantify how far the true probability of failure value was from the confidence interval provided by each stacking alternative. This metric revealed that the best linear weighting scheme was the Hierarchical Risk Parity method (HPR), despite the relative quality of the other stacking strategies proposed in the present paper.

Sincerely,

Luan Carlos de Sena Monteiro Ozelim, D.Sc.
Corresponding author on behalf of all authors
